# Persistent ascending mesocolon: an unexpected content in a giant and complex paraumbilical hernia of a 48-year-old obese woman

**DOI:** 10.1093/jscr/rjae834

**Published:** 2025-01-07

**Authors:** Nuhu N Naabo, Raymond S Maalman, Aishah F Adamu, Mohammed N Naabo, Samuel Mensah

**Affiliations:** Department of Surgery, School of Medicine, University of Health and Allied Sciences, PMB31, Ho, Ghana; Department of Surgery, Ho Teaching Hospital, P.O. Box MA 374, Ho, Ghana; Department of Basic Medical Sciences, School of Medicine, University of Health and Allied Sciences, PMB31, Ho, Ghana; Department of Basic Medical Sciences, School of Medicine, University of Health and Allied Sciences, PMB31, Ho, Ghana; Department of Health Promotion Sciences, University of Arizona, Tucson, Arizona 85721, United States; Department of Surgery, School of Medical Sciences, Kwame Nkrumah University of Science and Technology, P.O. Box Up 1279, Kumasi, Ghana

**Keywords:** paraumbilical hernia, persistent ascending mesocolon, obese, colon, appendix

## Abstract

Persistent ascending mesocolon (PAM) is a rare congenital anomaly in ⁓2%–4% of individuals. PAM is associated with various complications, including volvulus of the colon and caecum, bowel perforation, intestinal obstruction, and adhesions. This case is reported on a 48-year-old woman who reported to the Ho Teaching Hospital specialist clinic with a 13-year history of initial painless and reducible paraumbilical swelling. Management was based on a surgical approach. The content of the hernia sac was unusual with a viable appendix, caecum, ascending and transverse colons inclusive, and ascending mesocolon was persistent. The repair was done through an anterior component separation technique. An onlay mesh repair was fashioned with a 30 cm × 30 cm polypropylene mesh. This case report highlights the complexities of managing a giant paraumbilical hernia in a morbidly obese patient with a PAM, a rare congenital anomaly.

## Introduction

Persistent ascending mesocolon (PAM) is a rare congenital anomaly in ⁓2%–4% of individuals [1]. Normally, in the fifth month of gestation during the final stage of intestinal development, the primitive dorsal mesocolon of the ascending and descending parts of the colon fuses with the parietal peritoneum [2]. PAM is associated with various complications, including volvulus of the colon and caecum, bowel perforation, intestinal obstruction, internal, peri-caecal or paracolic hernias, and adhesions [1, 3–6]. Risk factors such as obesity, constipation, heavy weightlifting, chronic cough, benign hyperplasia of the prostate, smoking, and diabetes increase the risk of the development and recurrence of ventral hernia in patients [7]. Here, we report a unique case of a giant paraumbilical hernia containing the appendix, cecum, ascending colon with a PAM and part of the transverse colon in an obese woman. This case highlights the importance of understanding anatomical variations and their potential impact on surgical practice, particularly in managing complex hernias.

## Case report

**Figure 1:**
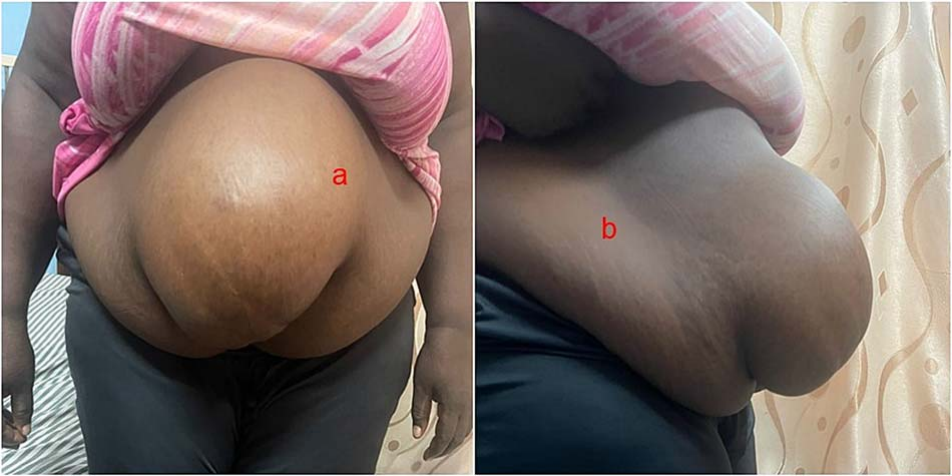
Showing the anterior (a) and lateral (b) views of the preoperative state of the woman.

This case is reported on a 48-year-old Ghanaian woman who reported to the specialist clinic of the Ho Teaching Hospital. She came with a 13-year history of initial painless and reducible paraumbilical swelling. Left untreated, it progressively increased in size and became partially reducible over 8 years with occasional colicky abdominal pain. On direct questioning, there was no history of chronic cough, constipation, or engaging in heavy manual work. She has no comorbidities like diabetes and hypertension. She, however, has a history of a previous caesarian section and left groin hernia repair. Physical examination revealed a morbidly obese woman (Body Mass Index = 50 kg/m^2^) with a reducible giant paraumbilical hernia with loss of abdominal domain (Fig. 1a and b).

In her current state, she visited three other health facilities with the same complaint, but surgery was not performed on two occasions due to her high body mass index. She defaulted on the third occasion, as the schedule coincided with an important family event.

An abdominal ultrasonography was done and it revealed a paraumbilical defect of ˃13.6 × 12.6 cm. A diagnosis of a giant complex paraumbilical hernia was made. Full blood count and renal function test were performed, and all the values were within the normal ranges for sex and age. The patient and her family were counseled about her condition, treatment modalities and the potential complications during and after the surgery. She underwent a successful anesthetic review, signed a consent form, and underwent surgery.

**Figure 2:**
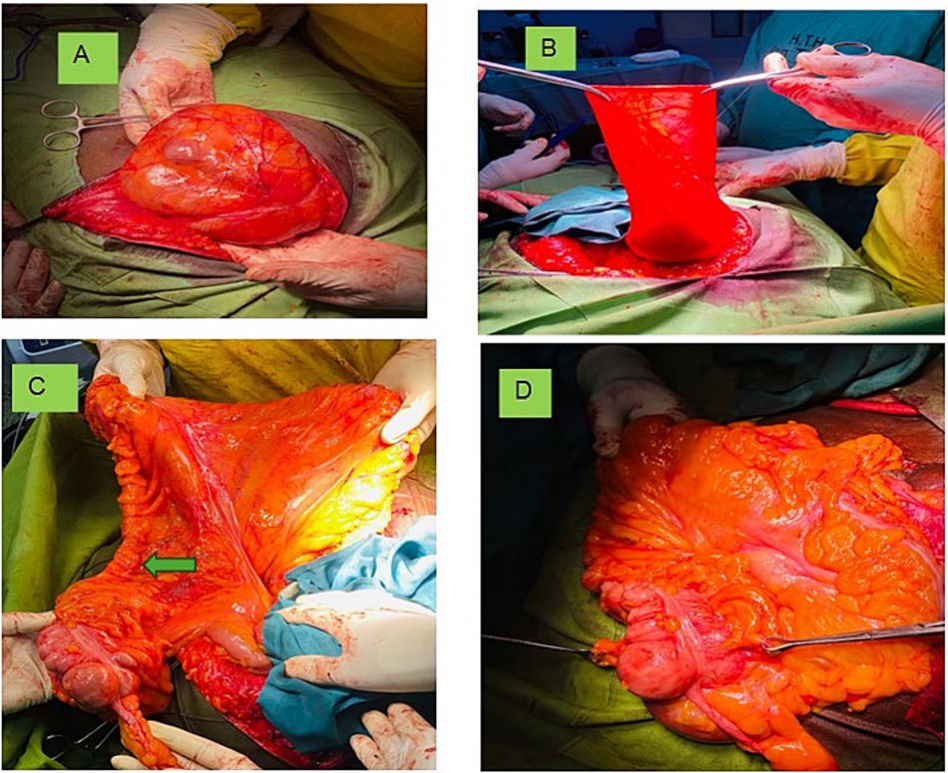
Showing (a) the incision unveiling the content covered with the sac; (b) the hernia sac; (c) the appendix (Blue arrow), PAM and colon (arrow head) continuing with the transverse colon; and (d) excessive fat on the mesentery.

An extended lower midline abdominal incision approach was adopted (Fig. 2a). The hernia sac was carefully dissected (Fig. 2b). The content of the hernia sac included a viable appendix, caecum, ascending colon, and part of the transverse colon which were separated, inspected and reduced (Fig. 2c and d). It was also observed that the ascending mesocolon was persistent making the caecum and ascending colon intraperitoneal instead of its usual retroperitoneum position. This makes the right colon freely mobile to form part of the hernia sac content of this case. Furthermore, it was found that the ascending colon was directly continuous with the transverse colon with no physical boundary as shown in Fig. 2c.

**Figure 3:**
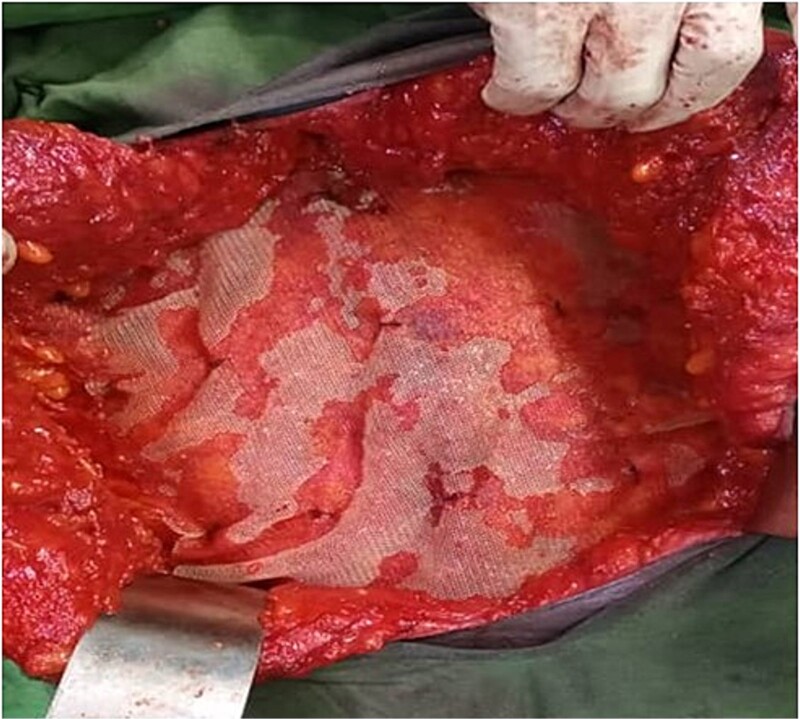
Showing the onlay 30 × 30 cm polypropylene mesh in-situ.

In addition, the defect size was huge and measured 22.5 × 20.5 cm and due to this huge defect and consequent lateralization of both rectus sheets, the anterior component separation technique was done to allow advancement of the rectus sheath into the midline for closure without tension. An onlay mesh repair was fashioned where a 30 × 30 cm polypropylene mesh was placed on the reconstructed abdominal wall (Fig. 3) and secured with nylon 3–0 sutures and the skin was closed with nylon 2–0. The patient developed a superficial surgical site infection post-surgery which responded to wound care and antibiotics. She was discharged subsequently.

## Discussion

This case was considered a complex condition with a high risk of post-operative complications as a result of the extremely high body mass index with a large defect of ˃10 cm. This might be the reason why the woman lived with the condition for several years and many surgeons were very careful to undertake such a high-risk surgical intervention. According to Slater *et al*. [7] major ventral hernias are characterized by defects ˃10 cm with significant contamination, or multiple complicating factors, and often require advanced surgical techniques like component separation techniques. Obesity is implicated in larger hernia size (>10 cm transverse width), an important risk factor for serious complications in ventral hernia repair. In addition, obesity leads to poor wound healing, thick subcutaneous fat, open surgery, more extensive dissection, longer operating times, wound drains, greater bleeding, dead space and risk of wound inoculation with bacteria, and decreased peri-operative subcutaneous tissue oxygenation [8].

The content of the hernia was also very unusual and included a viable appendix, caecum, ascending colon with a PAM and part of transverse colon. PAM, results in a long mesentery that permits the ascending colon to move medially, thereby vacating the right iliac fossa [1, 9]. The resulting altered spatial relationships, increased tension and potential weaknesses in the abdominal wall increase the risk of herniation [5] as seen in this case.

The huge hernia defect led to lateralization of both rectus sheets requiring an anterior component separation technique. This was done by an incision of the external oblique aponeurosis and the posterior rectus sheet on both sides, advancement of the rectus sheath into the midline and closure without tension. In ventral hernias where the rectus muscles cannot be approximated during repair, anterior or posterior component separation techniques are usually employed [10] that, surgeons with less experience more often favored the posterior component separation technique, whereas experienced surgeons preferred the anterior component separation technique.

Finally, the post-operative wound infection rate after onlay mesh repair for ventral hernia ranges from 11% to 15% [11]. The infections are widely treated by antibiotic regimens with or without percutaneous drainage, or by mesh removal [10]. Our patient only had a superficial surgical site infection which responded to wound care and antibiotics.

## Conclusion

In conclusion, this case report highlights the complexities of managing a giant paraumbilical hernia in a morbidly obese patient with a PAM, a rare congenital anomaly. The patient's unusual anatomy and significant morbid obesity made the surgery challenging, requiring advanced techniques like anterior component separation. This case emphasizes the importance of understanding anatomical variations, careful preoperative planning, and tailored surgical approaches to manage complex hernias effectively.

## Conflict of interest statement

The authors declare that they have no competing interests.
